# Thermoacoustic streaming in a linear temperature gradient

**DOI:** 10.1103/rn1j-19q5

**Published:** 2025-08-01

**Authors:** Enrico Corato, David van Assche, Ola Jakobsson, Wei Qiu, Per Augustsson

**Affiliations:** Department of Biomedical Engineering, Lund University, Lund, Sweden

## Abstract

Thermoacoustic effects arise when a temperature gradient is present in a sound field. This work investigates the interplay of orthogonal sound and thermal fields in a water-filled microchannel. We measured the three-dimensional streaming in the water-filled cavity for only sound applied, for only thermal gradient, and for the two fields combined. The resulting thermoacoustic streaming was 30 times faster than natural convection and 15 times faster than acoustic streaming. We also performed two-dimensional simulations of the channel’s cross section, reaching good qualitative agreement with the measured streaming data. Moreover, we measured the temperature of the fluid both with and without a sound field, showing that the thermal resistance of the channel decreased when the acoustic field was present, while we could not capture this in the current model. We believe our results can have implications for acoustically aided heat exchangers with liquid media.

## I Introduction

Acoustic streaming is the steady flow that typically originates from the attenuation of sound waves in the bulk of the fluid or in the viscous boundary. This type of streaming is commonly referred to as *dissipative*, since it relies on the viscous dissipation to generate forces that drive the fluid recirculation. Applications of acoustic streaming span a wide range of fields. It is important for sonic cleaning [1], surface chemistry [2], electrodeposition [3], and it has a crucial role in thermoacoustic engines [4,5]. Applied to bioanalytical assays, it has been employed to enrich extracellular vesicles [6,7], lyse biological membranes [8], rotate cells or small organisms [9], water atomization [10], enhance drug distribution by micropumping [11], and to achieve effective micromixing [12].

The understanding of acoustic streaming has grown since the first observations by Savart [13] and Faraday [14], and the first theoretical explanation by Lord Rayleigh [15], who gave a detailed description of the time-averaged steady flow in the fluid, generated by the oscillating velocities parallel to the boundaries. Schlichting [16] expanded this theory by describing the streaming inside the viscous boundary layer at the immediate proximity of the walls, which is driven by steep velocity gradients due to the no-slip condition at the cavity boundaries. Thus, in the case of a resonant standing wave, the inner boundary (or Schlichting) streaming can be seen as a slip velocity that powers the bulk (or Rayleigh) streaming. Acoustic streaming arises also in the case of a sound wave traveling in the bulk of the fluid for several wavelengths, as the energy is dissipated due to the fluid viscosity [17]. This net flow, termed Eckart streaming, occurs in the fluid bulk, away from the boundaries. Finally, Lighthill [18] generalized the acoustic streaming driving mechanisms as a nonzero divergence of the time-averaged acoustic momentum-flux-density tensor.

When medium inhomogeneities are present in a sound field—such as gradients in material properties caused by gradients of solute molecules—they result in a nonzero divergence of the time-averaged acoustic momentum flux density tensor. This nondissipative mechanism leads to acoustic body forces acting along the material gradients that can drive fast relocation of fluids [19–21] such that the fluid of highest acoustic impedance is brought to the pressure nodes of the acoustic field [22]. Given that diffusion is slow compared to this fluid relocation within a microcavity with gradients of density and compressibility, the re-organization drives the fluid into a stabilized state in which boundary-driven Rayleigh streaming is greatly suppressed [23,24]. Nevertheless, Schlichting streaming is still present and, together with the inevitable mass transport via diffusion, it contributes to the dissipation of the molecular gradient at long time scales. Eventually, the streaming transitions to characteristic Rayleigh streaming once the gradient has disappeared [25]. Inhomogeneity-induced streaming suppression allows for acoustic manipulation of submicrometric particles, such as bacteria [26,27], by tuning the flowrate to prevent the decay of the molecular gradient within the retention time in the microchannel.

An alternative way to generate acoustic body forces is to apply a temperature gradient in the acoustic cavity, since temperature affects both density and compressibility of a fluid. Such thermoacoustic effects have been extensively investigated for gases in various enclosures, such as cylinders [28,29] and narrow gaps between parallel plates [30]. These phenomena are closely related to thermoacoustic engines [5] and classic problems in combustion engineering, such as Rijke tubes [31], and have also been studied in more extreme conditions, including superfluid helium [32] and hot plasma [33]. Recently, the interaction between temperature and acoustic fields has been theoretically described for gases [34,35], with good qualitative agreement with experimental data [36]. In addition, this interaction and its effect on heat transfer has been studied numerically [37–39] and experimentally [40–42]. In contrast to the studies at macroscopic scale with a gas medium, we showed in a previous study that the thermoacoustic streaming induced by temperature gradients due to light absorption in a water-filled microchannel starts to play a significant role with a temperature gradient as small as 0.5 K/mm [43]. The experimental observations are well explained by the theory of the acoustic body force, and in excellent agreement with the streaming field calculated with an effective thermoviscous boundary layer [44]. Moreover, this effect has been shown to be influenced by the relative heat conductivities of the materials surrounding the fluid cavity [45]. Overall, the study of the interplay between acoustics and heat has significance for its potential application for acoustically aided heat exchangers [46,47]. To build such devices, where there is a sustained temperature difference across the fluid chamber, the interaction between acoustic and thermal fields must be better understood, especially concerning fluids with high heat capacity, such as liquids.

In this work, we investigated the interplay between a linear temperature gradient and a standing half-wave field perpendicular to each other in a water-filled microchamber. The thermal gradient was maintained by actively controlling the temperature on each side of the microfluidic chip, while the standing wave was excited by a piezoelectric element glued at the bottom. We experimentally characterized the temperature and streaming fields, and we compared qualitatively the observed and anticipated streaming fields through a numerical model. We obtained thermoacoustic streaming 30 times faster than natural convection, while the acoustic streaming was barely observed for a uniform temperature field. The thermoacoustic streaming could deform the linear temperature gradient, thus showing that it can facilitate the heat transfer in the microfluidic channel.

## II Materials and Methods

### A Setup

**Fig. 1:**
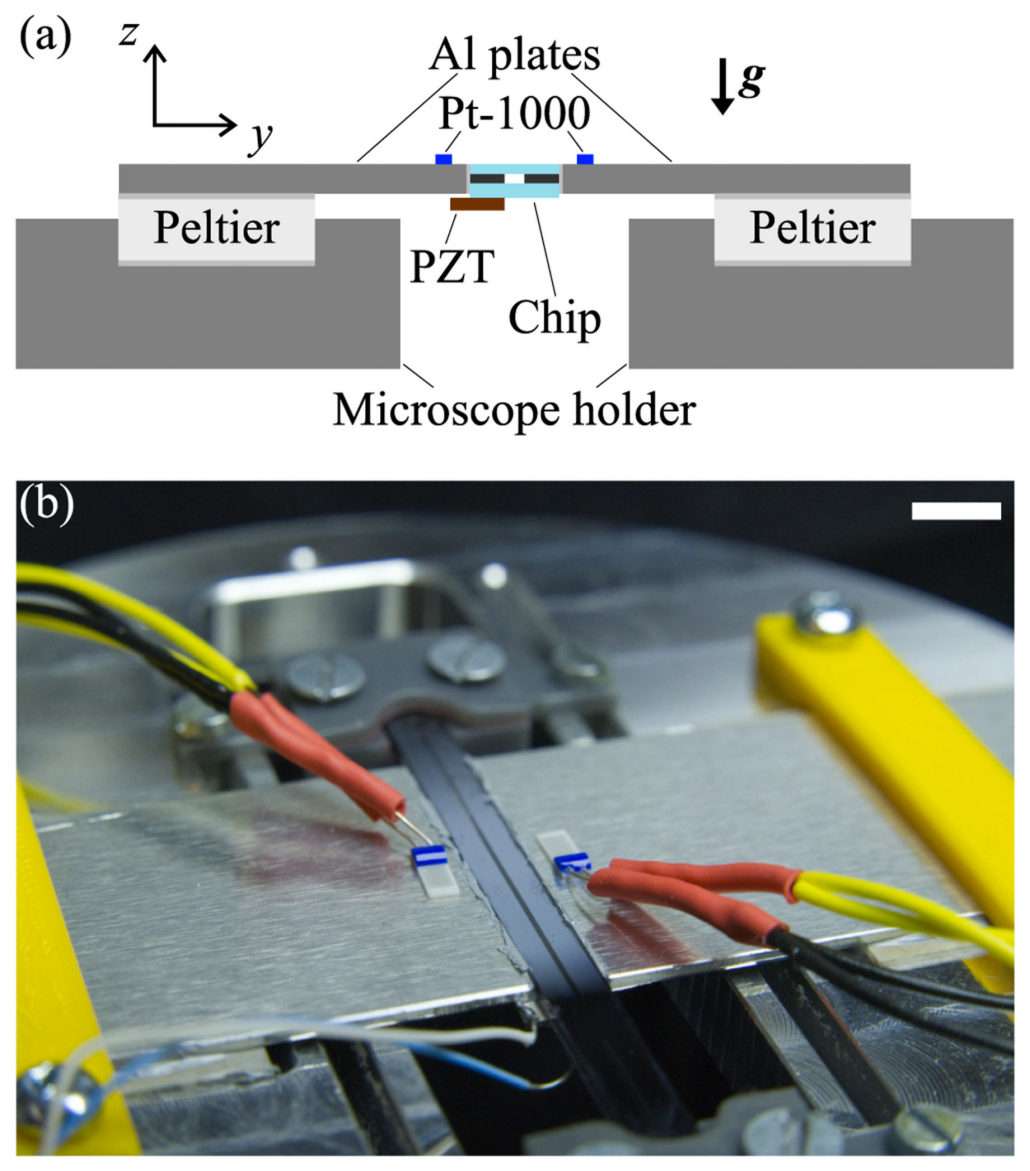
Setup used for all experiments. (a) Schematic of the cross section of the setup (not to scale). A thin layer of thermal paste (light gray) assured good thermal contact between all interfaces. The direction of gravity is indicated by ***g***. (b) Close-up picture of the chip assembly. Scale bar in the top right is 5 mm.

Figure 1 shows the chip assembly. A glass-silicon-glass microfluidic chip (GeSim Bioinstruments and Microfluidics mbH, Germany) was used in the experiments. It is comprised of a 375-μm-wide channel etched all the way through a 150-μm-thick silicon layer, which was then bonded to glass on both top and bottom of thicknesses 500 and 760 μm, respectively. Holes in the thicker glass allowed for fluidic inlets and outlets. The channel is 60-mm-long and centered in the 4-mm-wide chip. Imaging was performed utilizing a fluorescent microscope (BX51WI, Olympus, Japan) with 10x objective (NA 0.30, WD 10 mm), exiting fluorescence via a LED light source (pE-4000, CoolLED, UK) and collecting the emitted light via a digital CMOS camera (Orca Fusion BT, Hamamatsu, Japan). A piezoelectric transducer (Pz26, Ferroperm, Denmark), with dimensions 0.43 × 3 × 25 mm^3^, was glued on the bottom in the middle of the chip length, protruding on one side, Fig. 1(a). It was actuated with a sinusoidal signal at 4.99 MHz to excite a half-wavelength standing wave along the channel height by using an arbitrary waveform generator (33220A, Agilent, USA). The electrical power applied to the piezoelectric element was 15 mW, monitored by employing probes for current (CT-1, Tektronix Inc., USA) and voltage (CT2703, Cal Test Electronics Inc., USA), and recording their values using a PicoScope (5442D, Pico Technology, Cambridgeshire, United Kingdom). The temperature on the side of the chip was regulated by a dual channel thermoelectric cooler (TEC-1123-HV, Meerstetter Engineering, Switzerland) driving two Peltier elements (CP393365H, Same Sky, USA), which were placed in thermal contact with the two sides of the device via custom-made 1-mm-thick aluminum plates, Fig. 1(a). Thin layers of thermal paste (MasterGel Pro v2, Cooler Master Europe B.V., The Netherlands) were applied to ensure good thermal contact and, thus, effective heat conduction between the interfacing parts. The temperatures of the aluminum plates were measured by gluing a resistance temperature detector (Pt-1000, JUMO GmbH & Co., Germany) on each of them, as close as possible to the chip, without touching the thermal paste. These were used by the temperature regulator to monitor and maintain the temperature at set values. A picture of the device mounted on a microscope holder is shown in Fig. 1(b).

### B In-channel temperature measurements

Rhodamine B (Acros Organics, Fair Lawn, NJ, USA) was used as thermo-sensitive fluorophore to monitor the temperature inside the microchannel, as in [43]. In a nutshell, by setting equal temperatures to both sides of the chip within the range of interest, pixel-wise calibration curves were obtained to link the fluorescence intensity to the set temperatures. Then, we used the inverse relation to infer the unknown temperature field from the images intensity once the gradients had been generated across the channel width. Only 85% (~320 μm) of the channel width was considered in these measurements to avoid optical wall effects, such as reflection or scattering, that would have affected the temperature measurements. With this setup, the temperature could not be resolved along the depth of the channel, as the fluorescence intensity is heavily influenced by the light from out-of-focus planes [43].

### C Particle tracking

Tracer fluorescent polystyrene (PS) particles, 500 nm in diameter (G500, Fluoro-Max, Thermo Fisher Scientific, Waltham, MA, USA), were employed to map the streaming in three dimensions. These particles are well-below the critical diameter below which the background-boundary-driven acoustic streaming will dominate the particle motion [48]. Since thermoacoustic streaming velocity tends to exceed that of the boundary-driven streaming, any particle motion due to radiation forces acting upon the particles can safely be neglected. Fluorescent PS particles of diameter 4.9 μm (G0500B, Fluoro-Max, Thermo Fisher Scientific, Waltham, MA, USA), well above the critical diameter, were used to map the acoustic field for the constant temperature configuration. A four-port, two-way manual diagonal valve (V-101D, IDEX Health & Science, WA, USA) assured no flow through the channel when images were acquired. We used the MATLAB-based software *DefocusTracker* [49,50] to extract the particles’ positions and velocities. This software relies on the defocused images of the particles to estimate the position along the imaging axis. A cylindrical lens (LJ1558RM, Thorlabs GmbH, Germany) was placed in the optical path to induce astigmatism for the images of the particles, aiding us in tracking the three-dimensional motion [50,51]. We repeated the data collection between 50 and 100 times for the different experiments, resulting in thousands of partial particle trajectories for each condition.

### D Simulations

We implemented a numerical model in COMSOL Multi-physics 6.2. A two-dimensional cross section of the fluidic section of the chip was modeled using the modules *Pressure Acoustics, Heat Transfer in Fluids*, and *Laminar Flow*. The *Pressure Acoustics* and *Laminar Flow* modules were coupled with the *Acoustic Streaming Boundary* and *Acoustic Streaming Domain* Multiphysics. The Acoustic Streaming Multiphysics couplings are an effective model which enables solving for the resulting flow field without resolving the viscous boundary layer.

The fluid parameters were modeled using the built-in parameters of water and the fluid was assumed incompressible. Gravity was implemented in *Laminar Flow* as a volume force in the negative *z* direction with a magnitude of −***g****ρ*, where ***g*** is the gravitational constant and *ρ* the density of the fluid.

Given a standing wave inside a microresonator, the acoustic body force can be expressed as [43]

(1) $$\begin{array}{l} f_{ac}=-\frac{1}{4}{\langle P1\rangle }^{2}\nabla k_{s,0}-\frac{1}{4}{\langle v1\rangle }^{2}\nabla \rho 0\\ \;=-\frac{1}{4}\left[{\langle p1\rangle }^{2}{\left(\frac{\partial k_{s}}{\partial T}\right)}_{T_{0}}+{\langle v1\rangle }^{2}{\left(\frac{\partial \rho }{\partial T}\right)}_{T_{0}}\right]\nabla T_{0}. \end{array}$$

Our model calculates the first-order pressure and velocity fields *p*_1_ and ***v***_1_ from *Pressure Acoustics*, the temperature *T* from *Heat Transfer*, and the compressibility *κ*_*s*_ and density *ρ* from the material properties. The resulting streaming from all the forces acting on the fluid is computed from *Laminar Flow*.

The model is limited to the fluidic domain and does not include the silicon-glass chip nor the Peltier elements for temperature control. Therefore, the temperature profile was modeled by setting the temperature at the left and right boundary to the experimentally measured values when sound was on or off. For sound off, *T*_left_ = 25 °C and *T*_right_ = 46 °C, and for sound on, *T*_left_ = 27.5 °C and *T*_right_ = 43.5 °C. The top and bottom walls of the fluid channel were thermally insulated.

Acoustic actuation was implemented as a boundary velocity in the *z* direction. We did so by including a Thermoviscous Boundary Layer Impedance at the domain boundaries with a velocity *d*_0_*ω*_0_ in the *z* direction. Here *d*_0_ is the displacement amplitude while *ω*_0_ is the actuation frequency. Hence, the sound field was generated by the vibration at the boundaries, while the Boundary Layer Impedance accounted for the viscous and thermal dissipation in the acoustic boundary layer. To generate a curved pressure field, asymmetry was introduced in the boundary velocity. By varying the vertical velocity amplitude along the *y* dimension of the top boundary following a parabola *d*_0_*ω*_0_(−2 × 10^−5^ × *y*^2^ + 1), with − *W/*2 (⩽*y* ⩽ *W/*2, a curved pressure field was generated in the fluid domain at 4.6 MHz. We chose these actuation parameters after exploring different combinations of parabolic velocities and actuation frequency, as shown in Fig. 7 of the Appendix. The parabolic vertical velocity and the off-frequency actuation were necessary to break the symmetry of the numerical model.

The amplitude *d*_0_ of the displacement for the vertical velocity of the thermoviscous boundary layer was adjusted to match the measured streaming velocity in the channel for the given temperature field. For the curved pressure field, a displacement of 1.75 nm at 4.6 MHz was implemented to match the experimental velocity with the temperature profile.

## III Results

### A Experimental

**Fig. 2:**
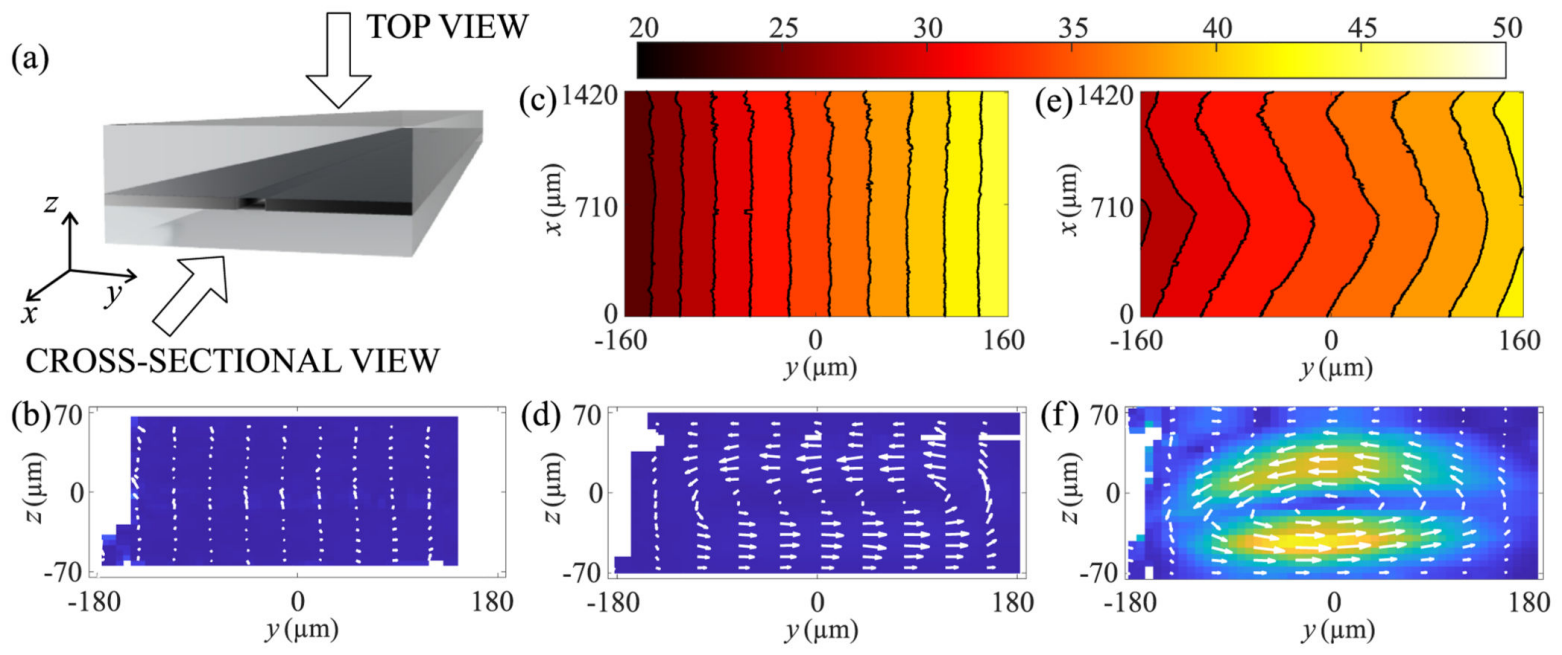
Measured streaming for different conditions, with velocity ranging from 0 μm*/*s (dark blue) to 160 μm*/*s (bright yellow), while voxels with missing data are shown in white. When a thermal gradient was applied, the measured temperatures in the channel are also shown. The unit of the temperature color bar is °C. (a) Rendering of the chip with top and cross-sectional views. (b) Streaming with only sound field condition, averaged along the whole length of the field of view. (c) Measured temperatures inside the channel with only thermal field condition Δ*T* = 30 °C. Isolines every 2 °C. (d) Streaming with only thermal field condition, as in (c), averaged along the whole length of the field of view. (e) Measured temperatures inside the channel with thermal and sound fields condition. Isolines every 2 °C. (f) Streaming with thermal and sound fields condition, as in (e), averaged along the whole length of the field of view.

To understand the thermoacoustic streaming generated by the interplay between the sound and temperature fields, we studied the effect of each field separately and of the two combined. Figure 2(a) defines the coordinate system and illustrates the top and cross-sectional view of the channel. When only the sound field was applied [Fig. 2(b)] we could detect very little motion inside the channel (average velocity of 11 μm*/*s) and the tracking algorithm had difficulties in tracking particles close to the side walls and, therefore, many voxels resulted in having no data. A temperature field was then applied by setting the two sides of the chip to 20 °C and 50 °C, respectively, resulting in a linear temperature gradient across the channel with a measured minimum of 24.8 °C and maximum of 45.9 °C [Fig. 2(c)]. The corresponding flow in the cross section is shown in Fig. 2(d). It follows the classic natural convection, with maximum velocity 5.6 μm*/*s, wherein gravity propels a flow due to the hotter fluid being less dense than the colder fluid. When both sound and temperature fields were applied, the temperature field [Fig. 2(e)] was clearly distorted by the thermoacoustic streaming, and the magnitude of the gradient decreased to a range between 27.5 °C and 43.5 °C. This indicates that the thermal resistance of the channel is somewhat decreased due to the enhanced heat transport by the generated thermoacoustic streaming. The transition between sound off and on can be seen in Movie of the Supplemental Material (SM) [52]. The resulting thermoacoustic streaming [Fig. 2(f)] is fast, with a maximum velocity ~160 μm*/*s. It consists of a big roll occupying most of the channel, with a small roll in the upper left corner. This was unexpected and it will be discussed in Sec. III B. The distorted temperature field along *x* can be explained by the varying streaming velocity along the length of the channel (Fig. 8 of the Appendix) likely due to a spatial variation in the acoustic field.

**Fig. 3:**
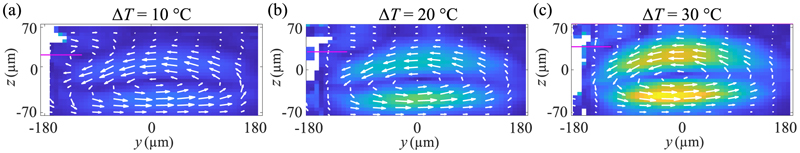
Thermoacoustic streaming for constant acoustic field and different temperature difference between the two side walls. The left wall was kept at 20 °C, while the right one was: (a) 30 °C, (b) 40 °C, and (c) 50 °C. The streaming velocity goes from 0 μm*/*s (dark blue) to 160 μm*/*s (bright yellow). The magenta line in each panel shows the center position of the top left roll, which is located at (a) *z* = 16.5 μm,(b)*z* = 24 μm, and (c) *z* = 34.3 μm.

We then characterized the thermoacoustic streaming for different temperature fields. Figure 3 shows the cross-sectional view of the streaming flow with the temperature sensors regulated to Δ*T* = 10, 20, and 30 °C, with corresponding maximum streaming velocity amplitude of 69 μm*/*s, 103.5 μm*/*s, and 156 μm*/*s, respectively. By increasing Δ*T*, besides the increasing velocity, we noticed that the top left roll got pushed more towards the ceiling of the channel.

**Fig. 4:**
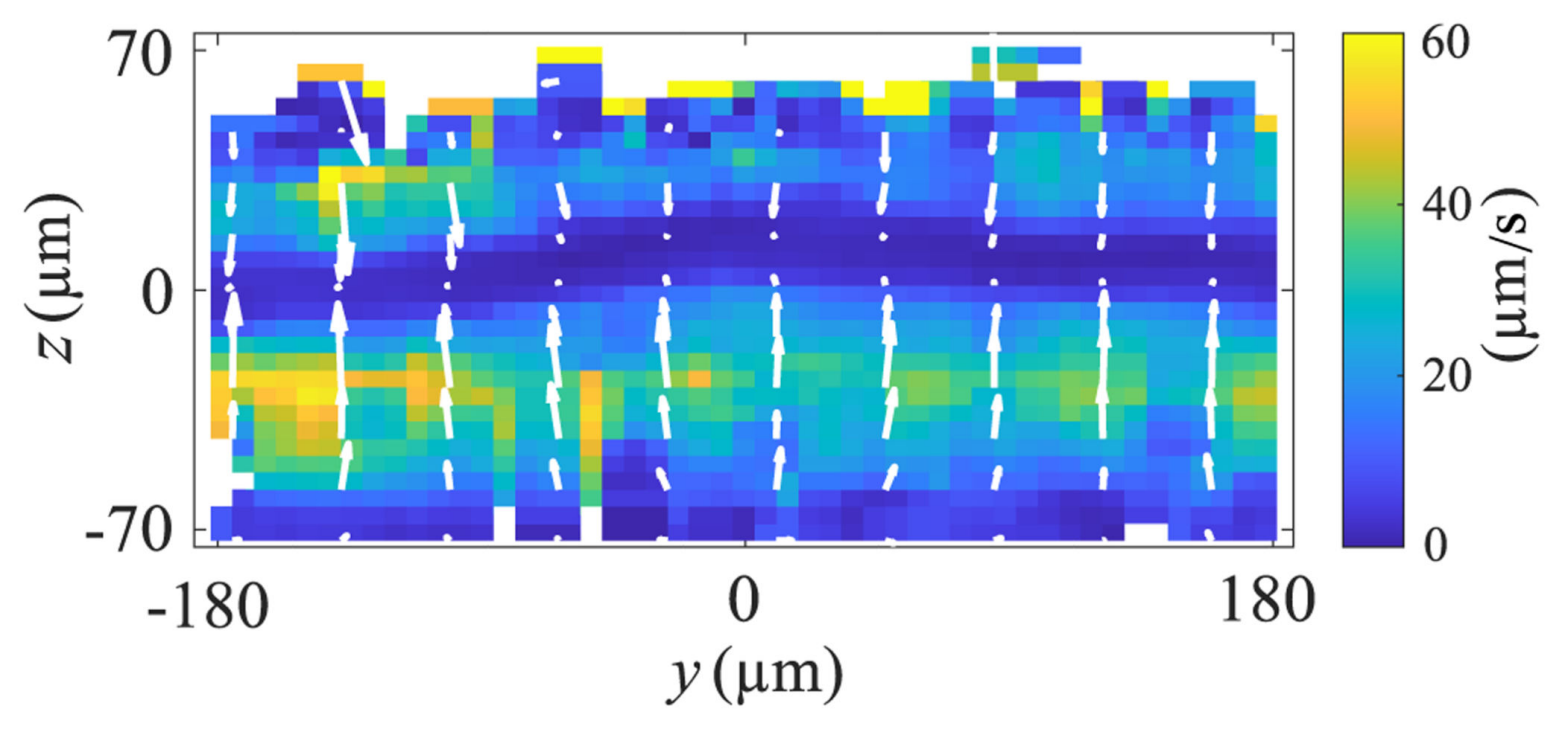
Cross-sectional view for the levitation of 5 μm PS particles averaged along the whole length of the field of view, with velocity ranging from 0 μm*/*s (dark blue) to 60 μm*/*s (bright yellow).

By tracking the motion of 5-μm-diameter PS particles, we were able to investigate the acoustic field inside the channel. Figure 4 shows the velocity field of such levitation without any temperature difference applied to the system. The pressure node, located near *z* = 0 at the channel mid-height, is clearly not straight, indicating that the sound field is not a plane standing wave. The curved pressure field, also slightly skewed towards the right, can explain the asymmetric thermoacoustic streaming we observed. This is further discussed in the following section, where two-dimensional numerical simulations are employed to illustrate how ideal and nonideal acoustic fields can affect the thermoacoustic streaming. The acoustic pressure amplitude (*p*_A_) can be roughly estimated from the acoustic radiation velocity *u*_rad_. By assuming an ideal plane wave and balancing the radiation force with the stokes drag force [48] we get $u_{\text{rad}}^{\max}=50\mu \text{m}/\text{s}$:

(2) $$p_{\text{A}}=\sqrt{\frac{6\eta u_{\text{rad}}^{\max}}{\Phi a^{2}k_{y}\kappa_{0}}}\approx 175\text{kPa}.$$

Here, *η* is the dynamic viscosity, Φ = 0.17 is the acoustic contrast of polystyrene particles in water, *a* is the particle radius, *k*_*y*_ is the wave vector, and *κ*_0_ is the compressibility of water.

### B Simulations

**Fig. 5:**
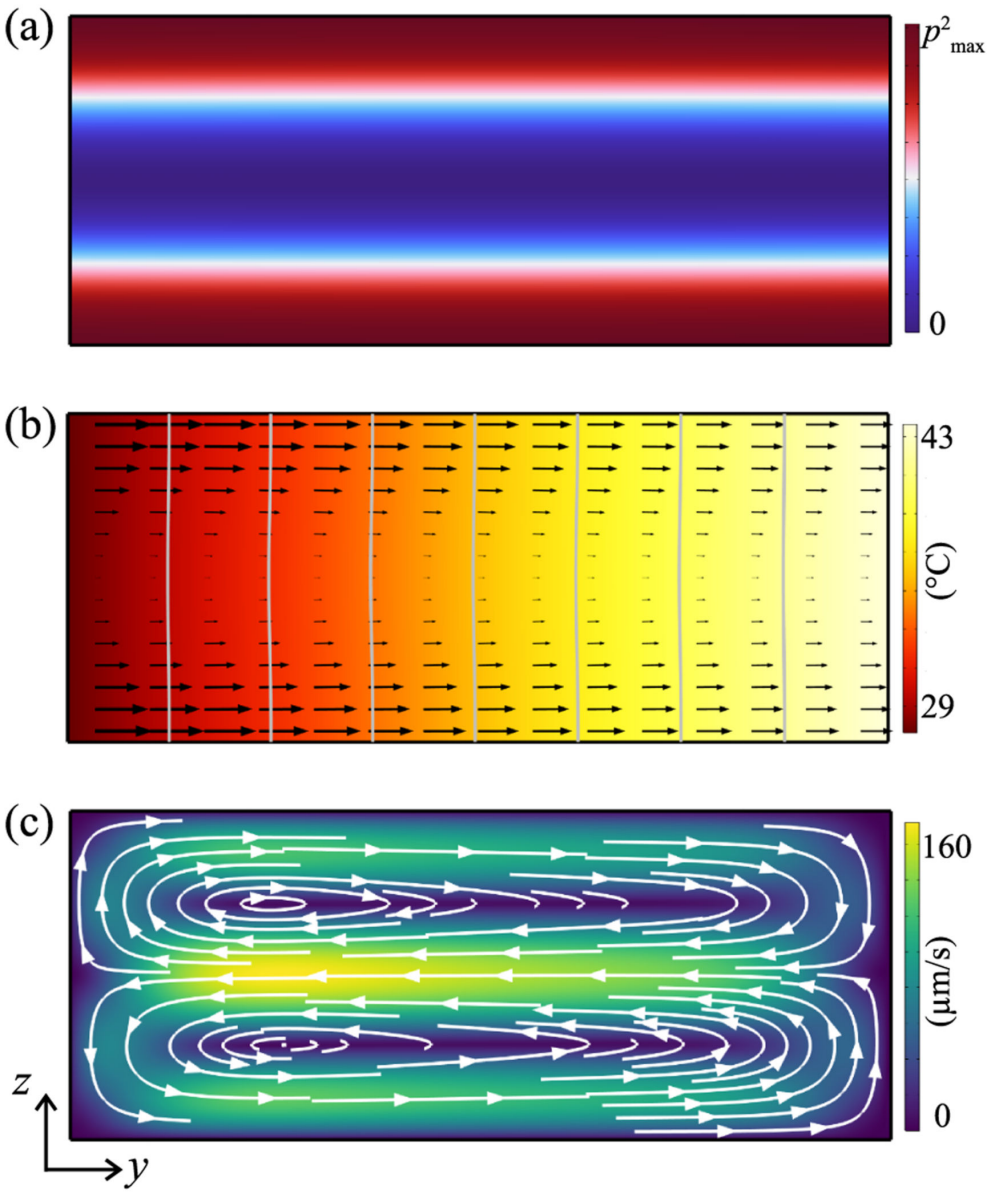
2D simulation of the channel cross section with ideal acoustic field. (a) Squared first-order pressure inside the channel, ranging from zero (dark blue) to maximum pressure (dark red). (b) Temperature distribution and acoustic body force (black arrows). The imposed Δ*T* was 16 °C, as measured in experiments, here shown from 27.5 °C (dark red) to 43.5 °C (bright yellow), with isolines every 2 °C (gray). (c) Thermoacoustic streaming across the channel, ranging from zero (dark blue) to maximum velocity (yellow).

We performed 2D numerical simulations to investigate the thermoacoustic streaming in an ideal and a nonideal standing-wave field. Figure 5 shows the case for an ideal acoustic field. Figure 5(a) shows the squared pressure field inside the water-filled channel in an acoustic plane wave with a pressure node at the mid-height. The temperature distribution is shown in Fig. 5(b), overlayed with arrows representing the acoustic body force, which is at its maximum near the pressure antinodes. The force is directed along the temperature gradient while the force magnitude primarily scales with the squared first-order acoustic pressure since the effect of the acoustic velocity field and the associated gradient in density is negligible. Looking at Eq. (1) the temperature field leads to both a density gradient and a compressibility gradient directed towards the cold side and associated body force components directed towards the hot side. But the components are not balanced, since for water ${\left(\frac{\partial \rho }{\partial T}\right)}_{T_{0}}\ll {\left(\frac{\partial \kappa_{s}}{\partial T}\right)}_{T_{0}}$ the thermoacoustic streaming is primarily driven by the generated compressibility gradient. The resulting thermoacoustic streaming, as shown in Fig. 5(c), is symmetric with respect to the pressure node, with the fluid recirculation pointing towards the hot wall at the pressure antinodes.

**Fig. 6:**
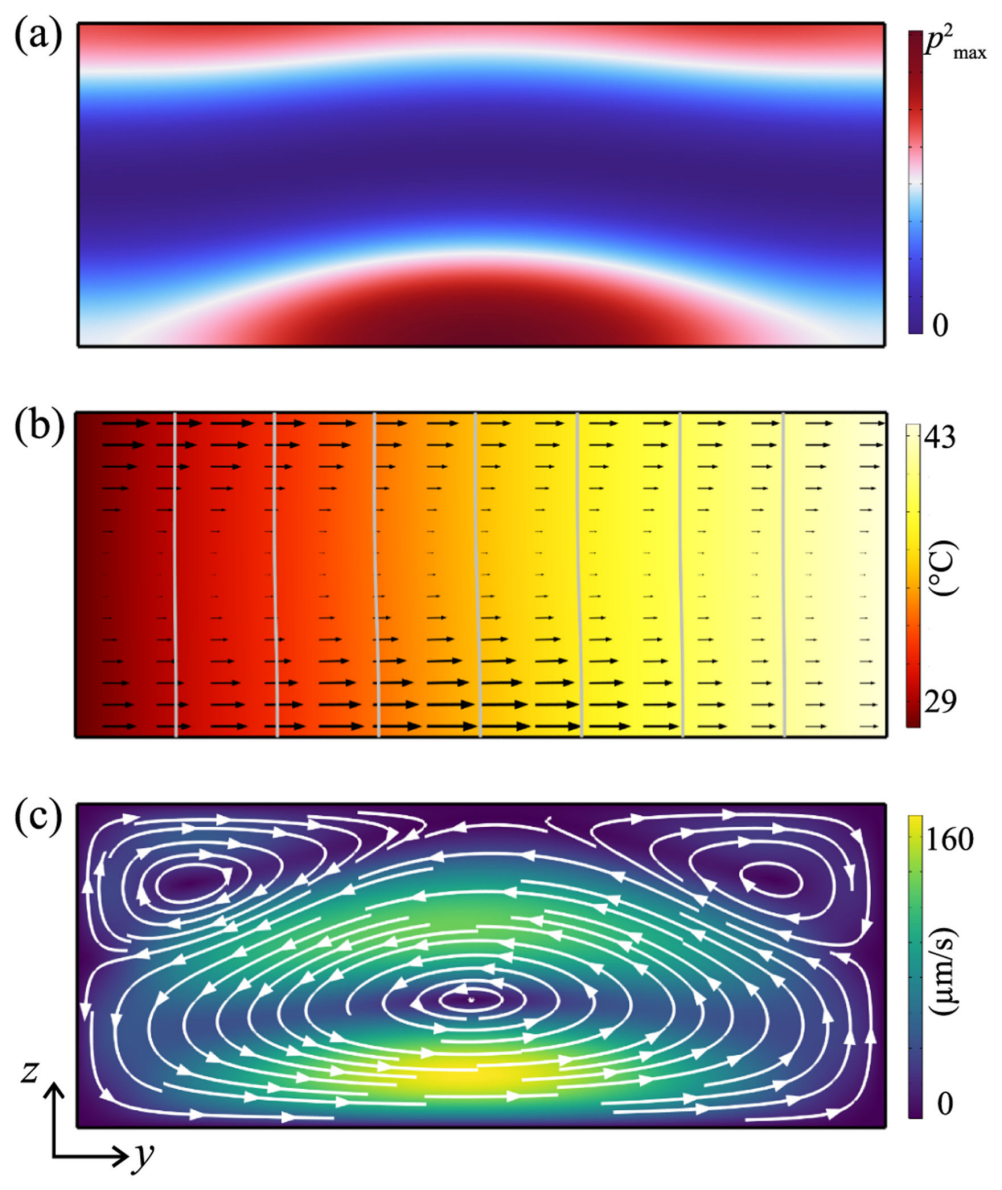
2D simulation of the channel cross section with curved acoustic field. (a) Squared first-order pressure inside the channel, ranging from zero (dark blue) to $p_{\max}^{2}=8\times 10^{10}{\text{Pa}}^{2}$ (dark red). (b) Temperature distribution and acoustic body force (black arrows). The imposed Δ*T* was 16 °C, as measured in experiments, here shown from 27.5 °C (dark red) to 43.5 °C (bright yellow), with isolines every 2 °C (grey). (c) Thermoacoustic streaming across the channel, ranging from zero (dark blue) to maximum velocity (yellow).

To understand the cause of the discrepancy between the experimentally observed streaming field [Fig. 2(f)] and the streaming field of the ideal system [Fig. 5(c)], we simulated the case of a curved pressure field (Fig. 6). In this simulation, the acoustic field is symmetric around *y* = 0 but not with respect to *z*, as indicated by the squared first-order pressure field [Fig. 6(a)]. This curved sound field, which aims to resemble the experimentally measured mode shown in Fig. 4, results in a different acoustic body force field [Fig. 6(b)] compared to the case with an ideal standing-wave field, even though the temperature field is identical in the two cases. The acoustic body force still points towards the hot wall at the pressure anti-nodes, but it is not symmetric with respect to the channel mid-height, as it is stronger close to the bottom. The resulting thermoacoustic streaming [Fig. 6(c)] is, thus, also not symmetric. The bottom roll is dominating the fluid recirculation, pushing the top roll against the ceiling of the channel and thus splitting it into two. Nevertheless, the vorticity of every roll remains coherent, namely with the flow pointing towards the hot wall at the pressure antinodes.

## IV Discussion

Thermoacoustic streaming is an inevitable effect that arises whenever a temperature gradient is present in a fluid-filled cavity with a sound field. This is important to consider for acoustofluidic applications, especially when driving the piezo-electric materials with high power, as they can heat up nonuniformly [53] and thus induce temperature gradients inside the fluidic chamber. With this work, we further validated the acoustic body force theory describing the interaction between sound and thermal fields in a water-filled microchannel. Despite not having an ideal acoustic standing-wave field in the channel, we could use numerical computation to compare the calculated and measured thermoacoustic streaming fields with good qualitative agreement. Looking in Fig. 6, we note that the simulated acoustic field that led to the best-matching thermoacoustic streaming field has a max pressure amplitude in the range 200 to 250 kPa, while our measured field has an estimated pressure amplitude of 175 kPa [Eq. (2)]; see Fig. 4.

We also measured the temperature inside the channel with and without the acoustic field. The temperature gradient decreased when the sound was turned on, indicating an increase in heat transfer across the channel. This could be of relevance for heat exchangers, as thermoacoustic streaming can improve the heat transfer without adding complexity in the fluid path. Hence, shorter loops and lower pressure drops could be employed in such acoustically aided heat exchangers. Combined with acoustic pumping [54], thermoacoustic streaming could be deployed in heat exchangers comprising no moving parts, making them more compact and, thus, suitable for cooling electronics in small probes for space exploration [55].

The simulations do not perfectly match the experimental results, since we could not capture the temperature change due to thermoacoustic streaming that we measured in experiments, as shown in Figs. 2(c) and 2(e). We can compare the timescales for thermal diffusion *t*_diff_ = *w*^2^*/*2*α* ≈ 0.5 s for a channel of width *W* = 375 μm and thermal diffusion constant *α* = 0.143 mm^2^*/*s, and for advective transport *t*_adv_ = *W/u*_str_. For only natural convection [Fig. 2(d)], *t*_adv_ ≈ 75 s and, thus, the temperature field can be assumed to be unaffected by the flow. For thermoacoustic steaming [Fig. 2(f)], *t*_adv_ ≈ 2.3 s with the highest measured velocity, which indicates that thermal diffusion should still be dominant over advective heat transport. However, we observed a quite-abrupt change in the temperature field in the channel, arising in the center of the channel and evolving towards the side walls (see Movie1 of the SM) [52]. This discrepancy could be caused by the temperature redistribution that we observed, which is a three-dimensional phenomenon that cannot be completely captured by a two-dimensional simulation (see Fig. 8 of the Appendix). Furthermore, the pressure field in our device is not only curved but is also skewed towards one side of the channel. Further studies are needed to overcome these limitations, for example, by computing the thermoacoustic streaming by using the measured acoustic field as an input for the simulation. Moreover, the numerical strategy to model fast thermoacoustic streaming should involve an iterative computational approach [56,57]. This is due to the modification of the equilibrium temperature distribution by streaming-induced heat transfer, occurring at a slow timescale, influencing the acoustic field, occurring at a fast timescale, which in turn further modifies the streaming. In addition, nonlinear effects in the thermo-viscous boundary layer could heavily influence the heat flux at the channel boundaries, making the finite-element model we used inaccurate. Previous studies focused on gaseous media [29], but our study indicates that further research is needed for liquids.

## V Conclusions

This paper has investigated the thermoacoustic streaming arising from the interplay of a linear temperature gradient orthogonal to a sound field in a water-filled microchannel. The experimental measurements showed that the fluid motion in the channel cross section with both sound and temperature fields was roughly 30 times faster than either natural convection or acoustic streaming. This clearly indicates that the nondissipative bulk-driven thermoacoustic streaming dominates over the dissipative boundary-driven Rayleigh streaming when temperature gradients are present in the system. We also showed how thermoacoustic streaming can affect the temperature field inside the channel, although the stationary simulation did not predict such an effect. Future work should focus on using iterative computational approaches to quantitatively match experiments and simulation. Based on our findings, we envision future applications of orthogonal thermoacoustic streaming for enhanced mixing in microfluidic systems and improved heat transfer in macroscale heat exchangers.

## Supplementary Material

Supplemental Material

Supplementary Materials

## Data Availability

The data that support the findings of this article are openly available [58].
