# Supplementary material for: Thermoacoustic streaming in a linear temperature gradient
Source: Phys Rev E. Author manuscript; Available in PMC 2025 Sep 24. (PMC7618165; doi:10.1103/rn1j-19q5)
Supplement: Supplemental Material [file EMS208794-supplement-Supplemental_Material.zip › Description_of_Supplementary_Materials.pdf]

Description of Supplementary Materials for

Thermoacoustic streaming in a linear temperature gradient

Enrico Corato, David van Assche, Ola Jakobsson, Wei Qiu, and Per Augustsson*
Department of Biomedical Engineering, Lund University, Lund, Sweden

* Communicating author: per.augustsson@bme.lth.se

Supplementary Video:

<Movie 1>
Temporal development of the temperature field in the thermoacoustic resonator after the onset of sound. The sound is turned on at ≈1.1 s.
