# Supplementary material for: Thermoacoustic streaming in a linear temperature gradient
Source: Phys Rev E. Author manuscript; Available in PMC 2025 Sep 24. (PMC7618165; doi:10.1103/rn1j-19q5)
Supplement: Supplementary Materials [file EMS208794-supplement-Supplementary_Materials.pdf]

APPENDIX

A nonideal sound field was introduced in the simulation by varying the boundary velocity and actuation frequency. Figure 7 shows the resulting pressure squared and thermoacoustic streaming for selected combinations of these two parameters. It is worth noticing that when the frequency is close to the ideal resonance along the channel height, i.e., 5 MHz, the pressure nodal line is always straight, thus leading to symmetric thermoacoustic streaming rolls. We chose the combination that leads to the streaming pattern that resembles the most the measured thermoacoustic streaming (Fig. 3), namely $f = 4.6$ MHz and $k = -2 \times 10^{-5}$.

The observed thermoacoustic streaming was a 3D phenomenon. Figure 8 shows how the streaming varies in the channel. Figure 8(a) depicts how the thermal gradient is affected by the different streaming velocities along the channel length x, showing the cross-sectional streaming in 10 sections. The streaming at different x-y planes along the height z of the channel is shown in Figs. 8(b)–8(d).

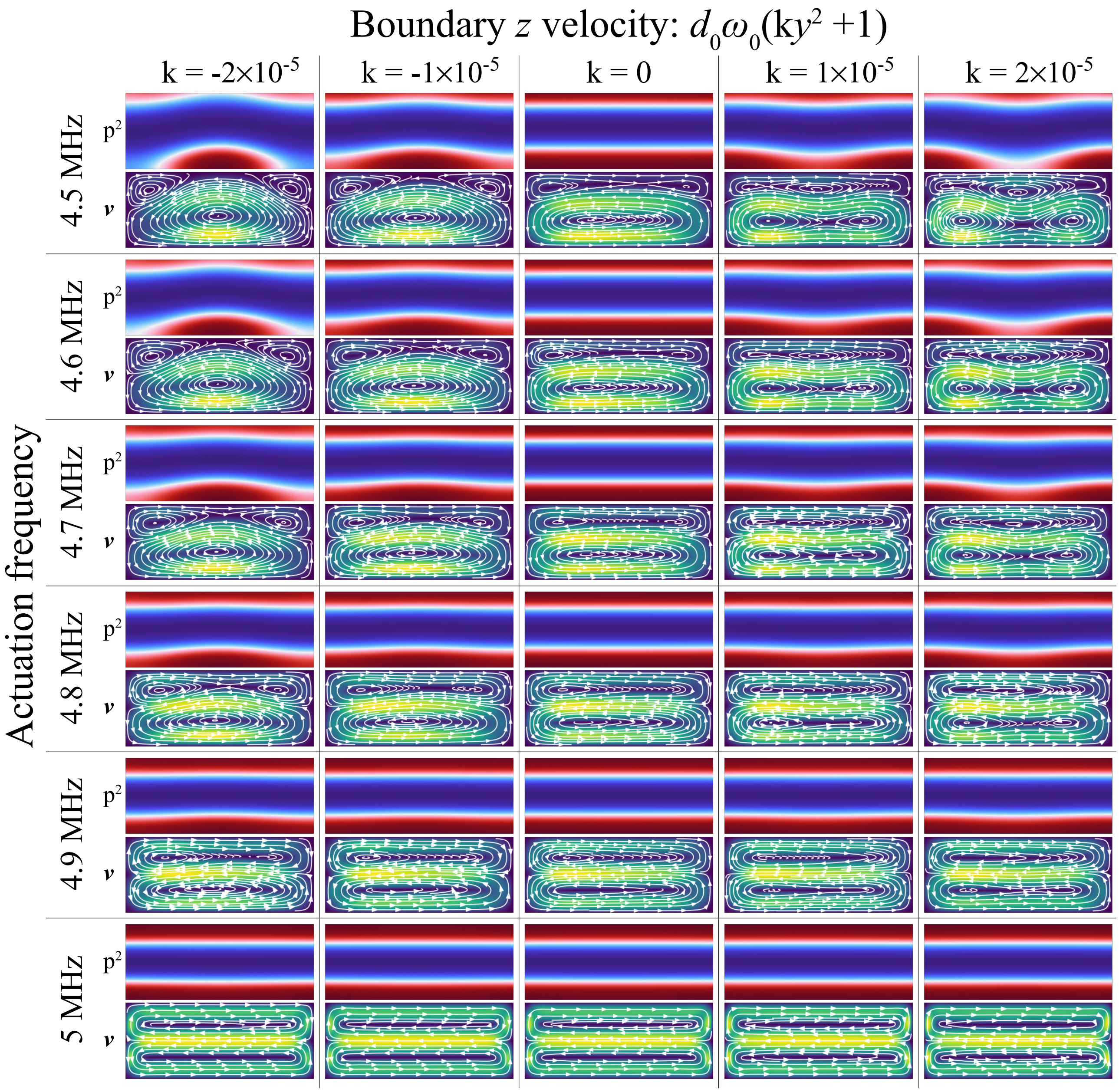

FIG. 7. Combinations of actuation frequency and boundary velocity for the pressure squared $\boldsymbol{p}^2$, from zero (dark blue) to maximum (dark red), and streaming velocity $\boldsymbol{v}$, ranging from zero (dark blue) to maximum velocity (yellow).

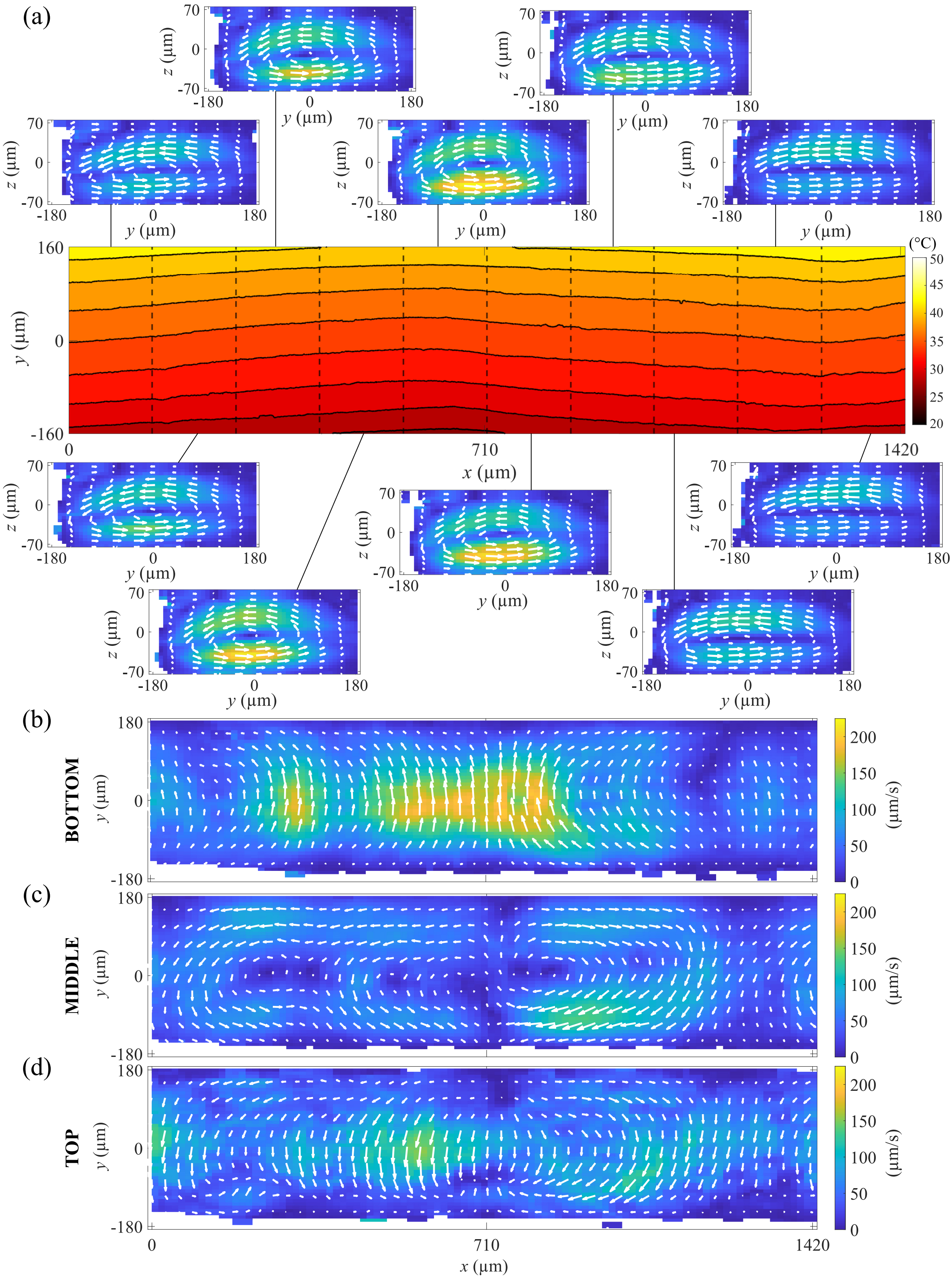

FIG. 8. (a) Thermoacoustic streaming in the y-z cross section extracted from 10 sections along x, with streaming velocity ranging from 0 (blue) to 225 μm/s (bright yellow). The measured temperature field for fully developed thermoacoustic flow is shown for reference, same as Fig. 2(e), with the positions of the 10 sections indicated by dashed black lines. Thermoacoustic streaming in the x-y planes extracted (c) at the bottom, (d) at mid height, and (e) at the top of the channel, each corresponding to a 50 μm slice in z.
